# Antioxidant capacity of human milk and its association with vitamins A and E and fatty acid composition

**DOI:** 10.1111/j.1651-2227.2009.01437.x

**Published:** 2009-11

**Authors:** A Tijerina-Sáenz, SM Innis, DD Kitts

**Affiliations:** 1 Food, Nutrition and Health, University of British ColumbiaVancouver, BC, Canada; 2 Department of Paediatrics, Child & Family Research Institute, University of British ColumbiaVancouver, BC, Canada

**Keywords:** Antioxidant capacity, Human milk, Polyunsaturated fatty acids, Vitamin A, Vitamin E

## Abstract

**Aim:**

The antioxidant capacity of human milk reflects the presence and activity of multiple components, which prevent oxidative rancidity. The aim of this study was to use the Oxygen Radical Absorbance Capacity assay to assess human milk antioxidant capacity and find correlations with milk components.

**Methods:**

Milk samples collected from 60 breastfeeding women at 1 month postpartum were assayed for antioxidant capacity, vitamins E and A, and fatty acids. Potential statistical relationships of concentrations of vitamins A and E and polyunsaturated fatty acids on the antioxidant capacity of human milk were determined.

**Results:**

Human milk antioxidant capacity was positively attributed to α-tocopherol concentration (ρ < 0.05). The vitamin A concentration did not significantly contribute to milk antioxidant capacity, but was correlated to milk α-tocopherol concentration (r=0.587; ρ < 0.001). There was no evidence of an inverse relationship between polyunsaturated fatty acids concentration and the antioxidant capacity value of milk.

**Conclusion:**

This study shows that α-tocopherol is an important contributor to the oxidative stability of human milk. Moreover, there was no evidence obtained to show that women who have high levels of milk polyunsaturated fatty acids are predisposed to lower milk antioxidant capacity.

## Introduction

Human milk is regarded as the ideal nutrient source for the growth and development of the infant, with unique composition characteristics distinct from both bovine milk and infant formula (1,2). Studies show human milk can suppress oxidative stress and oxidative DNA damage in newborn infants more effectively than infant formula and indicate that human milk contains a unique defence mechanism, which is not available in commercial infant formulas or in bovine milk (3). The antioxidant capacity (AC) of human milk comprises numerous bioactive components with varying capacities for antioxidant activity and provides stability towards onset of lipid oxidation reactions. For example, vitamins E and C, retinol and β-carotene, lactoferrin and glutathione, and antioxidant enzymes including catalase, superoxide dismutase and glutathione peroxidase are all present in human milk (3–5), and are known to have specific antioxidant roles against lipid peroxidation (6,7).

Milk with a higher AC value will reflect greater oxidative stability and a potentially greater protection for the breastfed infant from exposure to oxidative agents. The Oxygen Radical Absorbance Capacity (ORAC) assay using β-phycoerythrin (β-PE) as the fluorescent probe, known as ORAC_PE_, has been used previously to determine the AC of human milk (8). A modification of the ORAC procedure using fluorescein (FL) as the fluorescent probe, ORAC_FL_, has been proposed to have distinct advantages to determine the AC in both food and biological samples because of its greater sensitivity and photostability (9). Studies on AC of both food and biological systems have reported potential interactions of different compounds possessing antioxidant activity (10,11).

In the present study, the nutritional composition of human milk samples was measured with respect to the concentrations of vitamins A and E and fatty acids, for the purpose of determining whether or not these milk components are related to human milk AC.

## Methods

### Background of participants

Study participants were identified from registration records at the British Columbia’s Women’s Hospital, Vancouver, Canada. Eligibility criteria included women between 20 and 40 years of age giving birth to a single full-term gestation (37–42 weeks). Exclusion criteria included any pregnancy complications: diabetes, cardiac and renal diseases, or known communicable diseases. Pregnant women who had known substance abuse, or had insufficient skills in English to complete the informed consent and understand study questionnaires were also excluded. Women following a vegan diet, with significant food allergies or intolerances that restricted the intake of food groups, or routinely took fish or other oil supplements were also not enrolled. Socio-demographic information, maternal age, ethnicity, education, family income, and use of vitamin and mineral supplement were also recorded.

### Human milk samples

Milk samples were collected from 60 breastfeeding women at 1 month postpartum. The women were instructed to collect 50 mL milk by interrupting their infant’s feeding about 5 min after beginning nursing, thus providing a sample of hindmilk. Samples were collected into prelabelled vials provided to each mother, and the mothers were instructed to place the sample in their home freezer immediately after collection. The samples were transferred frozen to the Nutrition Research Laboratory at the Child and Family Research Institute (CFRI) and stored at −80°C until analysis.

### Milk vitamins A and E analysis

Vitamin A, all-*trans*-retinol, and isomers of vitamin E, α-tocopherol (α-Toc), δ-tocopherol (δ-Toc) and γ-tocopherol (γ-Toc) in human milk were analyzed by liquid chromatography (HPLC) following fat extraction (12). An aliquot of 500 μL of human milk was placed in a glass tube, 400 μL of 12% pyrogallol added, the sample vortexed and then dissolved in 1 mL distilled water and 600 μL ethanol, and re-vortexed. The extraction was repeated twice. A total of 4 mL hexane was added, mixed and centrifuged (2000 × ***g*** for 10 min). The organic layer was recovered, an aliquot of 3 mL taken and evaporated under nitrogen. Vitamin content was determined using an Agilent 1100 HPLC (Agilent Technology 1100 series, Palo Alto, CA, USA) with Chemstation software, column Phenomenex SphereClone 5u ODS (2) (5 μm, 150 × 4.6 mm) (Phenomenex Inc., Torrance, CA, USA). The mobile phase was 100% methanol at a flow rate of 1.3 mL/min and run time of 8 min. Detection wavelengths for α-Toc, δ-Toc and γ-Toc, and all-*trans*-retinol were set at 292 nm, 298 nm, and 325 nm respectively. Vitamin A and E contents in human milk were calculated using regression equations of calibration curves of known standards, with recoveries for the tocopherols (α-Toc, δ-Toc, γ-Toc) and all-*trans*-retinol all greater than 98.9 ± 1.1%, based on the addition of known quantities of pure standards to human milk.

### Milk fatty acid analysis

The composition of human milk fatty acids was determined as previously reported in detail (13). Briefly, 100 μL aliquot of human milk and 500 μg of heptadecanoic acid (17:0) as the internal standard were directly methylated in methanol–benzene (4/1, v/v) using acetyl chloride at 100°C for 30 min, and vortexing every 5 min. Then, 6 mL of saline and 3 mL of pentane was added, methyl esters recovered in the pentane phase, and the pentane extraction repeated twice. The fatty acid methyl esters were separated and quantified by GLC using a 30 m × 0.25 mm ID, 0.20 μm film nonbonded, fused silica capillary SP 2330 column (Supelco, Belefonte, PA, USA), with helium as the carrier gas at a column flow of 1 mL/min and inlet pressure of 15 lbs per square inch, with the inlet splitter set at 10 to 1. The samples were injected at 80°C, then after 2 min, the column oven temperature was increased to 170°C at 20°C/min, held for 25 min, heated to 195°C at 20°C/min. The injector and detector were set at 240°C and 260°C respectively. The milk fatty acid unsaturation degree was calculated as the unsaturation index (UI%), equivalent to the sum of the total number of double bonds (∑ (*m*_*i*_ × *r*_*i*_)), derived by multiplying the number of double bonds (*m*_*i*_) by the percent of the respective milk fatty acid (*r*_*i*_).

### Milk antioxidant capacity analysis

The antioxidant capacity (AC) of human milk was analyzed by the ORAC assay using fluorescein (FL) as the probe (9). The limits of assay precision, accuracy and linearity using the ORAC_FL_ procedure for human milk were previously determined as 2.2%, 94.8 ± 3.2% and R^2^ = 0.990 ± 0.005 respectively (14). Trolox and milk samples were previously dissolved in phosphate buffer (PB) to a final Trolox concentration of 0.0–4.0 μM and milk dilution of 200×. Fluorescein sodium salt (FSS) was added to a volume of 160 μL. The microplate was shaken for 10 sec and incubated to reach 37°C. The peroxyl radical initiator 2,2′–azobis (2-amidinoproprane) dihydrochloride (AAPH) was immediately added to all wells of the plate, except for those of the control and the blank. The final volume of the assay was 200 μL. The microplate was re-shaken for 10 sec, and fluorescence was read every minute for 60 min in a Fluoroskan Ascent FL (Labsystems) fluorometer at excitation of 485 nm and emission of 527 nm. Data integration and calculations were performed according to the method of Cao and Prior (15). Areas under the curve (AUC) of Trolox and human milk samples were calculated: 
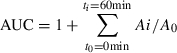
 where *A*_*0*_ is the initial fluorescence reading at 0 min and *A*_*i*_ is the fluorescence reading at time 60 min. Milk samples and Trolox net AUC values were obtained by subtracting the AUC value of the blank from that of the milk sample or from that of Trolox. Milk samples and Trolox net AUC values were plotted vs concentration to obtain the slope from regression analysis curves. The ORAC_FL_ value of a milk sample was calculated by dividing the slope of sample by the slope of Trolox. The final ORAC_FL_ values for human milk were expressed as an equivalent of the micromole concentration of Trolox solution per millilitre of milk (μmol TE/mL milk). Analyses were performed in triplicate.

### Ethics

The study protocol was approved by the University of British Columbia’s Clinical Screening Committee for Research and Other Studies Involving Human Subjects and the British Columbia Women’s Hospital Research Coordinating Committee. All participants provided written informed consent.

### Statistical analysis

Results are presented as mean ± SEM; n=60 for all measures. Distribution curves of milk test variables measured were normalized using the logarithmic transformation and potential relationships between human milk AC value and contents of vitamins A and E, polyunsaturated fatty acids (PUFA), and unsaturation index (UI%) were determined by the General Linear Model. Normalized distribution curves of milk variables (α-Toc, all-*trans*-retinol, total ω-6 PUFA, total ω-3 PUFA, total PUFA, UI% and AC) were divided into tertiles of low (*I*), medium (*II*) or high (*III*) contents. Tertiles were calculated by using standardized z-scores and the mean and standard deviation (±SD) of the milk variables. An odds ratio analysis was used to determine any potential effect of low, medium and high contents of α-Toc, all-*trans*-retinol, total ω-6 PUFA, total ω-3 PUFA, total PUFA and UI% on increasing the AC values of human milk. All statistical analyses were performed using SPSS© (SPSS Inc., version 10.0; Chicago, IL, USA) and Excel 2002 (Microsoft®, Redmond, WA, USA). The level of significance to detect statistical differences was set at ρ < 0.05.

## Results

The mean age of the 60 women at delivery was 33.4 ± 0.5 years, range 25–40 years, with 73% of Caucasian background, and 73% of the subjects also reporting that they had completed university or college training. All women reported taking multivitamin supplements. Our study population is thus more likely to reflect women with good nutritional care.

The average milk AC value was 3.41 ± 0.07 μmol TE/mL (Table 1). From the tocopherols, α-Toc was the major isomer found in milk with a mean of 2.32 ± 0.11 μg/mL milk, while γ-Toc and δ-Toc represented 19.8% and 4.7%, respectively, of the total milk tocopherols. All-*trans*-retinol was also present, but in relatively small amounts. The average milk fat content of samples collected from different subjects was 3.12 ± 0.12% (Table 1).

**Table 1:** Vitamin A and E, fat contents and antioxidant capacity of human milk*

Human milk variable	Concentration
all-*trans*-retinol (μg/mL)	0.08 ± 0.01 (0.01–0.20)
α-Toc (μg/mL)	2.32 ± 0.11 (0.66–5.02)
δ-Toc (μg/mL)	0.11 ± 0.01 (0.00–0.56)
γ-Toc (μg/mL)	0.46 ± 0.03 (0.11–1.27)
AC (μmol TE/mL)	3.41 ± 0.07 (2.26–4.90)
Fat (%)	3.12 ± 0.12 (3.02–3.87)

AC = Antioxidant capacity.

* Values represent mean ± SEM, (range in parenthesis), n=60.

The levels of fatty acids and total milk fatty acid unsaturation index (UI%) are shown in Table 2. Saturated fatty acids represented 39.0 ± 0.67%, monounsaturates 38.9 ± 0.56%, and total PUFA 16.8 ± 0.48%, with 14.7 ± 0.42% as ω-6 and 2.1 ± 0.09% as ω-3 PUFA in the milk fatty acids, as g/100 g. The UI of human milk was 77.5 ± 1.20%.

**Table 2:** Fatty acid composition and unsaturation index in human milk*

Fatty acids	g/100g FA
Capric (10:0)	0.87 ± 0.04 (0.24–2.0)
Lauric (12:0)	4.9 ± 0.24 (1.3–13.5)
Myristic (14:0)	6.2 ± 0.25 (2.7–13.8)
Palmitic (16:0)	20.3 ± 0.38 (14.7–26.2)
Stearic (18:0)	6.4 ± 0.20 (4.0–14.8)
Palmitoleic (16:1ω-7)	2.7 ± 0.09 (1.0–4.4)
Oleic (18:1ω-9)	35.3 ± 0.56 (21.0–45.6)
LA (18:2ω-6)	13.4 ± 0.41 (8.5–22.8)
AA (20:4ω-6)	0.42 ± 0.01 (0.26–0.58)
ALA (18:3ω-3)	1.5 ± 0.07 (0.4–3.4)
EPA (20:5ω-3)	0.09 ± 0.01 (0.00–0.38)
DHA (22:6ω-3)	0.30 ± 0.02 (0.08–1.11)
UI (%)	77.5 ± 1.20 (58.0–99.7)

LA = linoleic acid, AA = arachidonic acid, ALA = alpha-linolenic acid, EPA = eicosapentaenoic acid, DHA = docosahexaenoic acid, UI = unsaturation index (calculated as ∑ (*m*_*i*_ × *r*_*i*_), where *m*_*i*_ is the number of double bonds and *r*_*i*_ is the relative content of the fatty acid).

* Values represent mean ± SEM, (range in parenthesis), n=60.

**Figure 1:**
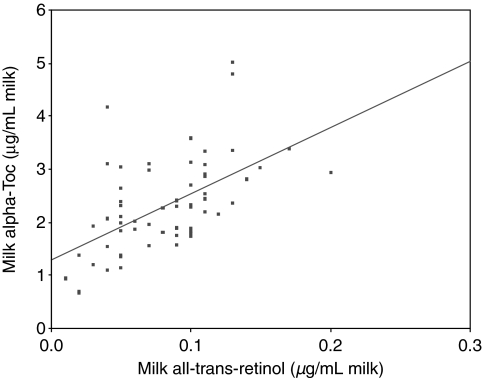
Relationship between concentrations of α-Toc and all-*trans*-retinol of human milk at 1 month postpartum, r=0.557 (ρ < 0.001), (n=60).

**Figure 2:**
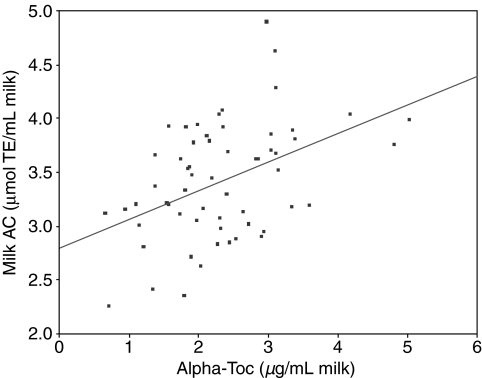
Relationship between AC value and α-Toc concentration of human milk at 1 month postpartum, r=0.439 (ρ < 0.01), (n=60).

Significant, positive correlations were found in milk between contents of α-Toc and all-*trans*-retinol, r =0.557 (ρ < 0.001) (Fig. 1) and milk AC value and α-Toc content, r =0.439 (ρ < 0.01) (Fig. 2). There were no significant relationships found between the milk AC value and all-*trans*-retinol content.

The odds ratios for different components in milk contributing to a high AC value are presented in Table 3. Despite the wide range of α-Toc concentrations in milk in the present study (0.66–5.02 μg/mL), α-Toc was the only tocopherol isomer that showed a significant (ρ < 0.05) contribution to the odds ratio for having a high milk AC value. We found no significant associations between the milk AC values and the levels of ω-6 or ω-3 PUFA, total PUFA, or the milk UI.

**Table 3:** Tertiles of milk variable values, odds ratio and 95% confidence interval for vitamin A and E, and unsaturated fatty acids, which relate to antioxidant capacity in human milk

	Tertiles of milk variable		
Milk variable	Tertile I (low)	Tertile II (medium)	Tertile III (high)
α-Toc (μg/mL)	1.51 ± 0.08 (0.66–1.93); 0.22 (0.07–0.75)	2.27 ± 0.04 (1.97–2.64); 1.15 (0.38–3.50)	3.34 ± 0.15 (2.71–5.02); 3.75 (1.22–11.55)**
All-*trans*-retinol (μg/mL)	0.04 ± 0.003 (0.01–0.06); 0.64 (0.22–1.90)	0.08 ± 0.003 (0.07–0.10); 0.62 (0.18–2.20)	0.12 ± 0.005 (0.10–0.20); 2.17 (0.75–6.29)
ω-6 LC-PUFA (g/100g FA)	1.04 ± 0.02 (0.73–1.17) 1.50 (0.51–4.40)	1.28 ± 0.01 (1.20–1.35) 0.64 (0.20–2.07)	1.49 ± 0.02 (1.38–1.69) 0.98 (0.34–2.87)
ω-3 LC-PUFA (g/100g FA)	0.33 ± 0.01 (0.17–0.41); 1.31 (0.46–3.87)	0.51 ± 0.01 (0.44–0.64); 1.06 (0.37–3.05)	0.90 ± 0.08 (0.65–1.74); 0.62 (0.18–2.20)
Total PUFAs (g/100g FA)	13.48 ± 0.24 (10.67–15.06); 1.06 (0.37–3.05)	16.74 ± 0.24 (15.40–18.24); 3.07 (0.97–9.75)	21.10 ± 0.64 (18.41–28.44); 0.34 (0.10–1.16)
UI (%)	67.94 ± 0.84 (58.03–73.25); 0.98 (0.34–2.87)	77.87 ± 0.49 (74.47–81.16); 0.81 (0.26–2.54)	87.68 ± 1.23 (81.54–99.69); 0.90 (0.30–2.70)

LC-PUFA = long-chain polyunsaturated fatty acid; UI = unsaturation index.

Data for each tertile include: Mean concentration ± SEM (range), n =60; OR (95% CI: 95% confidence interval).

** Denotes significance at p < 0.05.

## Discussion

In this study, human milk was collected at 1 month postpartum when lactation was well established. We focused our analysis to hindmilk because this contains about three times more fat than foremilk, and because the present study addressed the relationship between the levels of unsaturated fatty acids, fat-soluble vitamins and the antioxidant capacity of the milk.

We report the concentration of fat-soluble vitamins in milk and show a significant positive relationship between α-Toc and all-*trans*-retinol. Previous studies have reported the effect of maternal dietary vitamin intakes and vitamin supplementation on human milk, showing that higher intakes results in a higher concentration of the respective vitamin in milk, regardless of the stage of lactation (16,17). The positive correlation between vitamin A and E in milk found herein was consistent with the self-reported multivitamin supplementation for all the women.

The primary isomer of tocopherol present was α-Toc, with mean concentration similar to those found in milk of lactating women from Spain (16) and Cuba (17). Of interest, α-Toc is also the primary tocopherol isomer in bovine milk, although the reported concentration of 0.2–1.8 μg/mL is markedly lower than in human milk. Previous studies have shown that the lipid associated with the milk fat globule membrane contains three times the tocopherol content than that present inside the fat globule; leading to the suggestion that tocopherol is very important for stabilizing milk globule membrane (18). Our analyses show that γ-Toc was a second major tocopherol isomer in human milk, present in concentrations about 20% that of α-Toc, as has been reported by others for human (19) and bovine milk (20). Although γ-Toc represents about 70% of vitamin E in typical North American diets, with major food sources being soybean, corn and other vegetable oils, and nuts (21), α-Toc was the major tocopherol isomer present in the milk samples herein.

The all-*trans*-retinol concentrations found in human milk from Canadian women in the present study are comparable to previously reported vitamin A contents in milk from Icelandic women (22); although, the concentrations of vitamin A are considerably lower than the reported concentrations of other the primary carotenoids present in human milk, such as lycopene, β-carotene and lutein (17,23). To avoid technical problems leading to poor recovery of vitamin A from samples that have undergone freeze-thaw cycles (6,24), we analyzed only samples that had been frozen, but never thawed and refrozen prior to analysis, and conducted extensive studies with addition of pure lipid-soluble vitamins to human milk to ensure a high co-efficiency of recovery.

There is limited information concerning milk antioxidant capacity (AC) assessed using the ORAC_FL_ assay. Studies by Alberti-Fidanza et al. using the ORAC_PE_ assay reported a positive, time-dependent relationship between maternal dietary antioxidant intakes and milk AC (8). In the present study, the AC of human milk was measured using the ORAC_FL_ assay, which uses the peroxyl radicals generated by decomposition of AAPH to assess the radical scavenging capacity of human milk components. The AC values of Canadian women at 1 month postpartum varied from 2.26 to 4.90 μmol TE/mL milk, giving AC values at least twofold higher than the range of values of 0.36–2.18 μmol TE/mL milk reported by Alberti-Fidanza et al., for mature milk collected at day 20 postpartum from Italian women (8). The notable difference in AC values is likely attributed to the difference in the choice of fluorescence probe used. The ORAC_PE_ assay employs β-phycoerythrin (β-PE), a fluorescent probe that is less photostable than fluorescein (FL) (9), and which also has the potential to form nonspecific protein-binding complexes that reduces reactivity (15).

Miranda et al. used the ABTS-scavenging assay (Trolox Equivalent Antioxidant Capacity, TEAC) to evaluate the AC of infant formulas and found higher AC values in infant formulas that contained relatively high concentrations of vitamins A, E and C (6). In the present study, the AC of human milk could be significantly, positively attributed to the milk α-Toc content, but not to the vitamin A content. The lack of any relationship with vitamin A may be related to the relatively low amounts of vitamin A in human milk at 1 month postpartum. In this regard, Macias and Schweigert reported vitamin A concentrations of 1.02 ± 0.56 μg/mL in colostrum, which declined to 0.33 ± 0.14 μg/mL in the first weeks of lactation (17) raising the possibility that vitamin A could contribute to human milk AC in early lactation.

It is well known that levels of ω-6 and ω-3 PUFA vary widely among individual women, a difference which is explained largely by the types of ω-6 and ω-3 PUFA in the breastfeeding mother’s diet (25–27). In this study, the relative low variability in total milk fat content between women is in part due to our sampling protocol as well as the similarities in maternal dietary fat intakes. The high levels of unsaturated fatty acids in milk, represented by calculation of the number of unsaturated double bonds, or UI, suggest a high susceptibility for lipid peroxidation and rancidity (6), both of which will determine the eventual stability of human milk to peroxidation reactions. However, the present study found no statistically significant effect of total PUFAs, individual ω-6 or ω-3 long-chain polyunsaturated fatty acids (LC-PUFA), or UI on the AC values of human milk. Based on our results, it is reasonable to conclude that mothers following usual dietary practices where linolenic acid (ALA, 18:3ω-3) comprises up to 3.4%, eicosapentaenoic acid (EPA, 20:5ω-3) up to 0.38% and docosahexaenoic acid (DHA, 22:6ω-3) up to 1.11% milk fatty acids do not predispose their milk to oxidative rancidity. More likely, human milk AC value represents a complex mixture of numerous compounds with antioxidant activities functioning by different chemical reactions (4,28,29), which collectively culminate in a stable food source for the breastfed infant.

In conclusion, the AC of human milk obtained from 60 breastfeeding women at 1 month postpartum was significantly attributed to the presence of milk α-Toc, emphasizing the importance of this particular antioxidant vitamin, a finding complementary to the important role of vitamin E as a radical scavenger in many other biological systems. The lack of significant effects of other milk components analyzed in this study as having a role in modifying the oxidative stability in human milk, may reflect the sum of antioxidant activity defined by other mechanisms of action that coexist to directly reduce the presence of free radicals. Finally, the present study suggests that usual maternal dietary intakes should not represent a risk for decreasing milk AC.
